# Postprandial blood glucose response to a standard test meal in insulin-requiring patients with diabetes treated with insulin lispro mix 50 or human insulin mix 50

**DOI:** 10.1111/j.1742-1241.2008.01850.x

**Published:** 2008-09

**Authors:** Y Gao, G Li, Y Li, X Guo, G Yuan, Q Gong, L Yan, Y Zheng, J Zhang

**Affiliations:** 1 Department of Endocrinology, Peking University First HospitalBeijing, China; 2 Department of Endocrinology, Sino-Japan Friendship HospitalBeijing, China; 3 Department of Endocrinology, The 2nd Affiliated Hospital of Sun Yat-Sen UniversityGuangzhou, China; 4 Eli Lilly Asia, Inc.Shanghai, China

## Abstract

**Aim:**

To compare the 2-h postprandial blood glucose (PPBG) excursion following a standard test meal in insulin-requiring patients with diabetes treated twice daily with human insulin mix 50 vs. insulin lispro mix 50 (LM50).

**Methods:**

This was a multicentre, randomised, open-label, crossover comparison of two insulin treatments for two 12-week treatment periods in 120 Chinese patients. One- and 2-h PPBG and excursion values were obtained following a standardised test meal. Fasting blood glucose (FBG), haemoglobin A1c (HbA1c), insulin dose, rate of hypoglycaemia and safety data were obtained. A crossover analysis using SAS Proc MIXED was employed.

**Results:**

Mean 2-h PPBG excursion decreased from 6.32 ± 3.07 mmol/l at baseline to 3.47 ± 2.97 mmol/l at end-point in the LM50 group, and from 6.31 ± 2.88 at baseline to 5.02 ± 3.32 mmol/l at end-point in the human insulin mix 50 group (p < 0.001). Two-hour PPBG (p = 0.004) and 1-h PPBG excursion (p < 0.001) were significantly lower with LM50 as compared with human insulin mix 50. Both treatment groups were equivalent for HbA1c control, 1-h PPBG and insulin dose requirements. Mean FBG was higher with LM50 than with human insulin mix 50 (p = 0.023). The overall incidence of treatment-emergent adverse events and hypoglycaemia rate per 30 days were similar between treatment groups.

**Conclusions:**

Insulin lispro mix 50 provided better postprandial glycaemic control compared with human insulin mix 50 while providing the convenience of injecting immediately before meals. Both treatments were generally well tolerated by all randomly assigned patients.

**What's known**

Premixed basal and prandial insulins provide relatively convenient and consistent dosing. Human-insulin mixtures, widely prescribed in China, have slower onset and longer duration of action than rapid acting insulin analogues such as insulin lispro. Several clinical trials have consistently reported better postprandial blood glucose (PPBG) control with fixed mixtures containing insulin lispro.

**What's new**

For the first time, rapid-acting insulin lispro and basal insulin lispro protamine suspension (ILPS) in a 1 : 1 ratio (LM50) was investigated against human insulin mix 50 in a twice-a-day regimen in Chinese patients with diabetes. LM50 demonstrated better postprandial but equivalent overall glycaemic control compared with human insulin mix 50 following a high carbohydrate meal representing a typical Chinese breakfast.

## Introduction

Glycaemic control is fundamental in the management of diabetes. The goal of diabetic therapy is to achieve fasting and postprandial glucose concentrations and haemoglobin A1c (HbA1c) as close to normal as possible without hypoglycaemia (1). Postprandial hyperglycaemia is an early abnormality in the progression of type 2 diabetes and is problematic in patients with fasting hyperglycaemia (2). According to Monnier et al. (3), the contribution of postprandial blood glucose (PPBG) excursions to overall hyperglycaemia (represented by HbA1c) is predominant in patients with moderate hyperglycaemia (HbA1c < 7.3%), whereas the contribution of fasting hyperglycaemia is greater and increases with worsening overall glucose control (HbA1c > 8.4%). These observations were expanded by Monnier et al. (4) who found that as HbA1c levels increase with duration of type 2 diabetes in patients not treated with insulin, diurnal glycaemic control is lost in progressive steps – first during postprandial periods, then in the morning period (during the ‘dawn phenomenon’ of rising blood glucose), and then in the nocturnal fasting period. Therefore, as glycaemic control improves with basal-insulin treatment, PPBG coverage is needed to achieve or to keep HbA1c at < 7%. In addition to being a marker for the onset of type 2 diabetes, elevated PPBG is an independent risk factor for the development of micro- and macrovascular complications and affects the morbidity and mortality associated with long-term hyperglycaemia (5–7).

According to the American Diabetes Association (1), individuals with premeal glucose values within the target but not meeting HbA1c targets should monitor for 1–2 h for PPBG and treat to reduce PPBG values to < 10 mmol/l. The International Diabetes Federation (8) recommends treating both PPBG and fasting blood glucose (FBG) at any HbA1c level to achieve and maintain optimum glycaemic control, targeting PPBG < 7.8 mmol/l.

Thus, PPBG measurement and control are important in overall diabetes management. PPBG profile is determined by multiple factors including carbohydrate absorption, insulin and glucagon secretion, and their coordinated glucose metabolism in the liver and peripheral tissues. Abnormalities in insulin and glucagon secretion, hepatic glucose uptake, suppression of the hepatic glucose production, and peripheral glucose uptake associated with type 1 and type 2 diabetes contribute to higher and more prolonged PPBG excursions than in individuals without diabetes. The timing of PPBG measurement is crucial to acquiring the best information. As the complete absorption of food from a typical meal takes 5–6 h, measurement of plasma glucose 2 h after the start of a meal approximates the peak PPBG value and provides a reasonable assessment of postprandial hyperglycaemia (2).

This study was conducted in China, where the number of people with diabetes or prediabetes has increased dramatically over the past two decades because of the growing economy and improved standard of living that has resulted in the consumption of a higher-calorie, higher-fat diet with more processed foods and a parallel reduction in physical activity (9). A typical Chinese diet, including rice and wheat breads, consists of approximately 58% carbohydrates, which can induce high PPBG, especially postbreakfast (10). Optimal post-prandial glycaemic control for these high carbohydrate meals may require higher doses of rapid-acting insulin than often used in other economic and cultural environments.

Evaluation of intensive insulin regimens has suggested that to achieve optimal glycaemic control, therapy with at least two insulin formulations differing in their time-activity profiles is required. Treatment with conventional human insulin mixtures, however, may result in a non-physiological blood glucose response to a meal with high PPBG excursions, an extended period of hyperglycaemia and the risk of hypoglycaemia later in the day (11). Rapid-acting insulin analogues (insulin lispro, insulin aspart and insulin glulisine) premixed with basal components overcome many of the limitations of regular insulin therapies because of faster onset and shorter duration. In several studies, these analogue formulations provided better or equivalent postprandial glycaemic control with a reduced risk of hypoglycaemia compared with premixed human insulin along with greater flexibility and convenience of injecting immediately before meals (11–14).

Insulin lispro mix 50 (LM50: Humalog® Mix50™, Eli Lilly and Company, Indianapolis, IN) contains 50% insulin lispro and 50% insulin lispro protamine suspension (ILPS). Insulin lispro, the rapid-acting component, addresses the insulin requirements related to the morning and evening meals, and ILPS provides basal insulin throughout the day and also during the night.

In this study, we compared LM50 with human insulin mix 50 (50% regular human insulin, 50% human insulin isophane suspension; Novolin® 50, Novo Nordisk, Bagsværd, Denmark) for the control of 2-h PPBG excursion following a standard test meal in Chinese patients with type 1 or type 2 diabetes. In addition, FBG, 1- and 2-h PPBG, 1-h PPBG excursion, HbA1c, insulin dose requirements and safety of the two formulations were also assessed.

## Methods

### Study design

**Figure 1:**
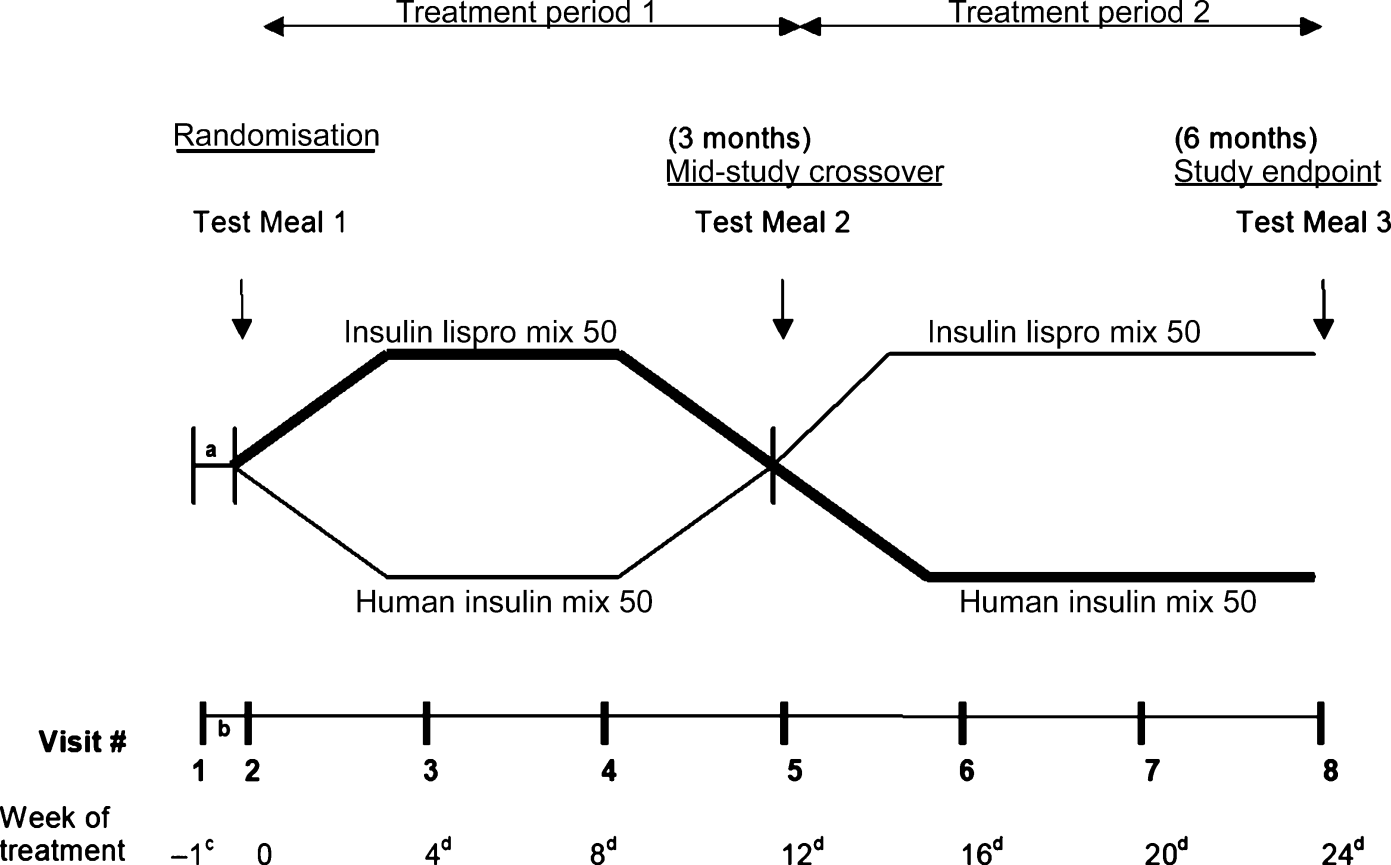
Study design: (a) lead-in period, (b) screening visit, (c) ±3 days and (d) ±7 days

This study was a multicentre (three centres in China), randomised, open-label, 2-sequence, 2-period, crossover trial in patients with type 1 or type 2 diabetes treated twice daily with human insulin mix 50 vs. LM50. Standard test meals were administered to compare these insulin treatments for their effect on 2-h PPBG excursion (Figure 1). The 2-h PPBG excursion was the blood glucose measurement 2 h after the start of the test meal minus the FBG measurement immediately prior to the test meal. Secondary objectives included FBG prior to the test meal, 1-h and 2-h PPBG and 1-h PPBG excursion following the test meal, changes in insulin dose requirements throughout the study and HbA1c at treatment end-points. The study protocol was approved by the local Medical Research Ethics Committee of all participating centres. The participants signed an informed consent document to participate in the study in accordance with the Declaration of Helsinki and good clinical practice guidelines.

### Patients and study conduct

One hundred and twenty patients of Chinese origin, with either type 1 or type 2 diabetes (aged 18–70 years) were recruited. Criteria for inclusion were the diagnosis of diabetes of at least 2 months’ duration, an HbA1c of 1.1–1.7 times the upper limit of the normal reference range (inclusive) and signed informed consent. The patients should have been using human insulin mix 50 or human insulin mix 30 twice daily as the only pharmacological treatment for their diabetes for at least 2 months prior to the enrolment in the study.

Individuals were ineligible if they had used oral antidiabetic agents within 60 days prior to recruitment, received a total daily dose of insulin > 2 units/kg, experienced two or more episodes of severe hypoglycaemia (requiring external assistance) within preceding 6 months, a body mass index > 35 kg/m^2^, serum creatinine more than the upper limit of normal, a history of class III/IV cardiac disease or renal transplantation, obvious clinical symptoms of liver disease, hepatitis, alanine transaminase more than three times the upper limit, known allergy to insulin or excipients contained in insulin products, or undergoing therapy for a malignancy other than basal cell or squamous-cell skin cancer. Female patients who were not practising a birth control method or were pregnant or breast-feeding were also excluded.

Enrolled patients were randomised to two groups in a 1 : 1 ratio, with 60 patients in each sequence group. One sequence group received 12 weeks of twice-daily treatment with LM50, followed by 12 weeks of twice-daily treatment with human insulin mix 50 (sequence 1). The other group received the reverse treatment of the sequence 1 (sequence 2).

At visit 1, patients were evaluated for their significant medical history including duration of diabetes, duration of insulin treatment, previous therapy for diabetes, current drug therapy and any pre-existing conditions to ensure consistency of inclusion and exclusion criteria. From visit 2 through visit 8, the efficacy and safety of LM50 compared with human insulin mix 50 in the treatment of diabetes were evaluated. Test meals were administered to patients at visits 2, 5 and 8 (Figure 1).

All insulin injections were given subcutaneously using insulin pens (HumaPen Ergo®, Eli Lilly, for LM50; NovoPen® 3, Novo Nordisk, for human insulin mix 50). Throughout the study, investigators adjusted the morning and evening dose of human insulin mix 50 or LM50 in accordance with the individual needs of the patient. LM50 and human insulin mix 50 may be administered at equivalent doses; however, patients were monitored for changes in insulin requirements following conversion between the two mixtures. Patients were educated regarding the time of insulin action for each insulin mixture. Human insulin mix 50 was administered within 30 min before the morning and evening meals. LM50 was administered within 15 min prior to the morning and evening meals. Patients performed self-blood glucose monitoring (at least daily testing of morning fasting serum glucose and once- or twice-weekly testing of PPBG was recommended).

The primary efficacy measure in this study was to compare the 2-h PPBG excursion after the test meal at visit 5 (study midpoint) and 8 (study end-point). Additional efficacy variables included 1- and 2-h PPBG, 1-h PPBG excursion from the test meal, FBG, HbA1c and insulin-dose requirements.

Patients consumed the standard test meal that comprised of 90 g noodles, representing a typical Chinese breakfast. The total caloric content of the standard test meal was 460 kcal (carbohydrates 65%, fat 20%, protein 9.3% and others 5.7%). Patients ate the same test meal at the same time of day at each of these visits, and the content of the test meal was identical for all patients at all study sites.

Patients were monitored for safety closely throughout the trial. Participants recorded any episodes of hypoglycaemia, defined as blood glucose < 3.5 mmol/l, accompanied by subjective symptoms or identified by signs considered to represent hypoglycaemia noted by an observer. At each patient visit, the rate of hypoglycaemia was computed as the number of hypoglycaemic episodes per patient adjusted for 30 days (episodes since previous visit/patient/30 days). Adverse events, concomitant therapies taken, laboratory data and vital signs of all randomised patients were also recorded at regular intervals.

### Statistical analyses

With an expected dropout rate of not > 15% after randomisation, at least 100 patients were expected to complete the study. The sample size for this study was selected to provide at least 80% power to achieve statistical significance if the true treatment difference was at least 1.0 mmol/l and was calculated using a two-sided one-sample *t*-test at α = 0.05.

Efficacy and safety analyses were conducted on the modified intent-to-treat population that included all patients randomised to one of the sequence groups and had received at least one dose of insulin therapy. In general, descriptive summary statistics was included for categorical and continuous variables. All comparisons were performed using two-tailed tests with a nominal significance level of 0.05.

The efficacy variables (2-h PPBG excursion, 1-h PPBG excursion, 2-h PPBG, 1-h PPBG, FBG and HbA1c) were analysed using the mixed model, with fixed effects for sequence, period, treatment, baseline FBG as a covariate, which was only adjusted for the analysis of PPBG excursion, and patient nested within sequence as a random effect (15). Analysis for binary response data suitable for the two-period crossover design was conducted using Prescott's test (16). For patients with a particular measurement missing at end-point (at last scheduled visit of each period), the most recently observed datum from the same treatment period was used in the analysis.

## Results

### Demographic and baseline characteristics

**Figure 2:**
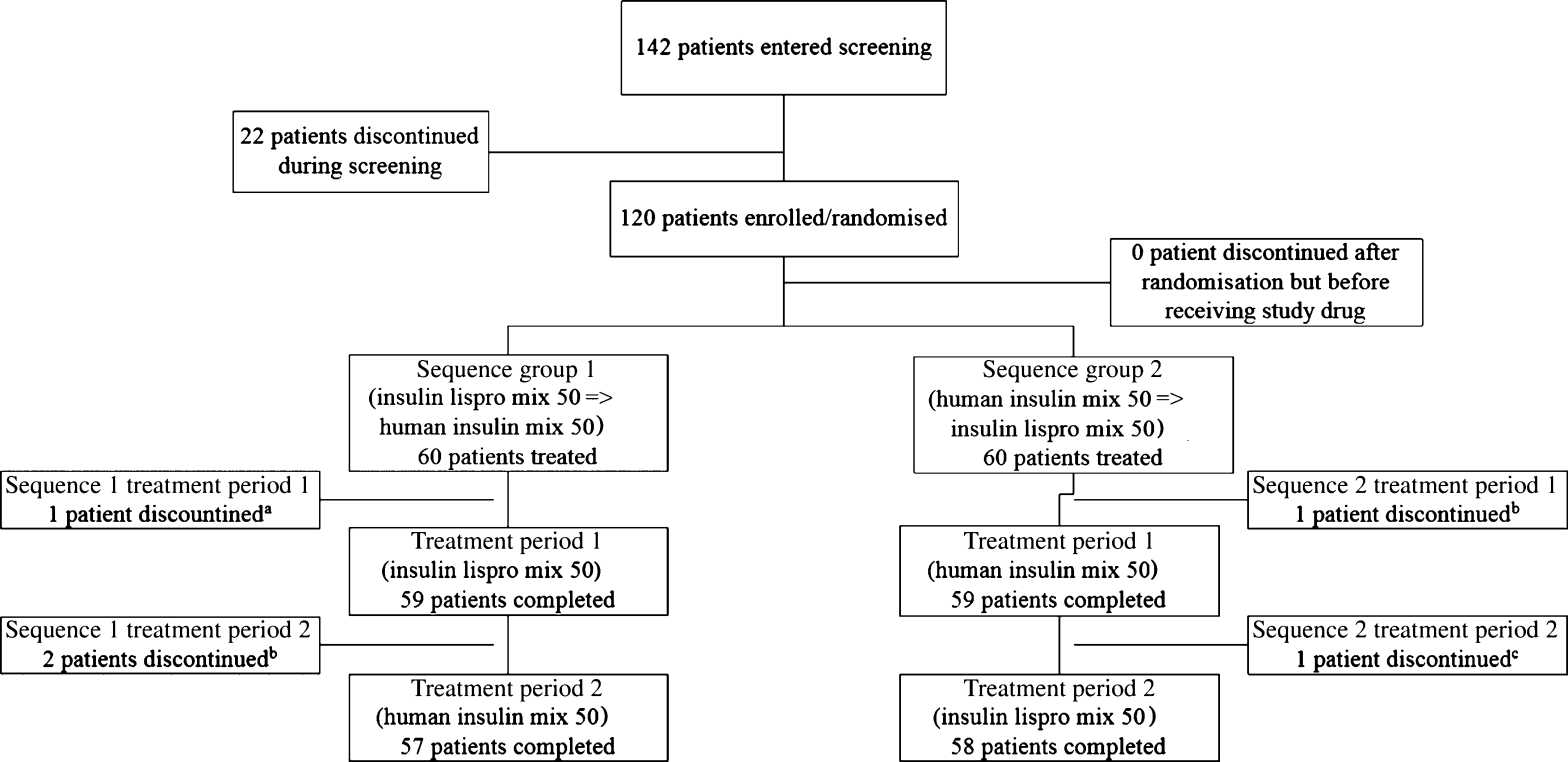
Patient disposition: (a) reason for discontinuation/non-compliance, (b) reason for discontinuation/adverse events and (c) reason for discontinuation/personal conflict or other patient decision

Of the 142 patients screened, 120 patients were included in the study, with 60 randomised to each sequence group. Table 1 summarises the demographic and baseline characteristics for all randomised patients (108 with type 2 and 12 with type 1 diabetes). One hundred and fifteen patients completed the study (57 in sequence group 1 and 58 in sequence group 2). Figure 2 presents a schematic representation of patient disposition in the study.

**Table 1:** Demographic and baseline characteristics (mean ± SD) for all randomised patients by treatment sequence

Variable	Statistic		
	Sequence group 1, lispro→human *N*=60	Sequence group 2, human→lispro *N*=60	Overall *N*=120
**Gender *n* (%)**			
Male	22 (37%)	26 (43%)	48 (40%)
Female	38 (63%)	34 (57%)	72 (60%)
Age (years)	54.3 ± 10.1	57.2 ± 8.6	55.7 ± 9.5
Body mass index (kg/m^2^)	24.5 ± 2.6	24.4 ± 2.8	24.5 ± 2.7
**Type of diabetes *n* (%)**			
Type 1	6 (10%)	6 (10%)	12 (10%)
Type 2	54 (90%)	54 (90%)	108 (90%)
Duration of diabetes (months)	142.2 ± 75.5	131.8 ± 73.0	137.0 ± 74.1
Duration of insulin treatment (months)	42.8 ± 55.9	41.2 ± 48.2	42.0 ± 52.0
HbA1c (%)	8.10 ± 1.38	8.03 ± 1.22	8.07 ± 1.30
Fasting blood glucose (mmol/l)	9.75 ± 3.22	9.35 ± 2.93	9.55 ± 3.07

*N*, sample size; *n*, number of patients; HbA1c, haemoglobin A1c.

### 2-h postprandial blood glucose excursion

Mean 2-h PPBG excursion decreased from 6.32 ± 3.07 mmol/l at baseline to 3.47 ± 3.00 mmol/l at end-point in the LM50 group (a reduction of 2.89 ± 3.27 mmol/l), and from 6.31 ± 2.88 mmol/l at baseline to 5.02 ± 3.32 mmol/l at end-point in the human insulin mix 50 group (a reduction of 1.32 ± 3.38 mmol/l). The decrease in 2-h PPBG was significantly greater with LM50 when compared to that with human insulin mix 50 (p < 0.001).

**Figure 3:**
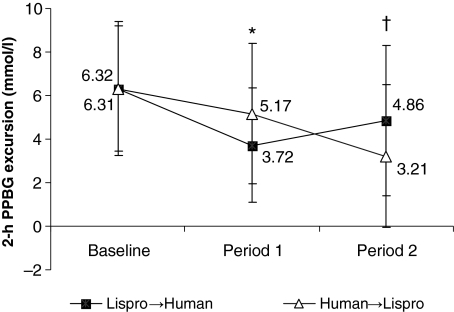
Mean 2-h postprandial blood glucose (PPBG) excursion at baseline and following treatment with insulin lispro mix 50 and human insulin mix 50, at end-point, by sequence group for each period, for all randomly assigned patients receiving at least one dose. *Statistically significant difference between sequence group 1 and sequence group 2 in period 1, p = 0.008. †Statistically significant difference between sequence group 1 and sequence group 2 in period 2, p = 0.010

Analysis of 2-h PPBG excursion at end-point by sequence group for each treatment period showed patients on LM50 had statistically significantly lower PPBG than those on human insulin mix 50. Treatment differences of similar magnitude at the end of each treatment period indicate no carryover between periods (Figure 3).

### Blood glucose

**Figure 4:**
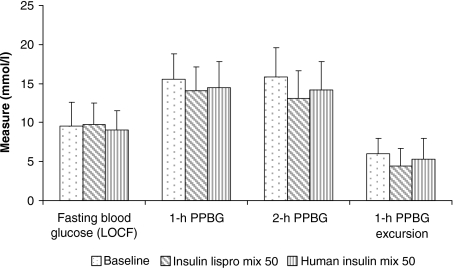
Blood glucose analyses, at baseline and following treatment with insulin lispro mix 50 and human insulin mix 50 at end-point, by treatment group for all randomly assigned patients receiving at least one dose. The difference between insulin lispro mix 50 and human insulin mix 50 was statistically significant for fasting blood glucose (FBG) (p = 0.023), 2-h PPBG (p = 0.004) and 1-h PPBG excursion (p < 0.001) but non-significant for 1-h PPBG (p = 0.253)

Figure 4 compares blood-glucose values at baseline and treatment end-points. Except 1-h PPBG, all other measurements were observed to be significantly different between treatment groups. The mean FBG was higher in patients on LM50 than in those on human insulin mix 50 (p = 0.023), whereas, 2-h PPBG (p = 0.004) and 1-h PPBG excursion (p < 0.001) were lower with LM50 than with human insulin mix 50.

### Haemoglobin A1c

The two treatments provided equivalent mean HbA1c values (p = 0.581) and mean change from baseline HbA1c values (p = 0.456) at treatment end-points. Mean HbA1c was 7.59% (decreased 0.48% from baseline) with LM50 and was 7.61% (decreased 0.46% from baseline) with human insulin mix 50.

### Insulin dose requirement

The insulin doses were similar in both treatment groups, at each visit and end-point, for morning, evening and total doses. The change from baseline in insulin dose between treatment groups was not significantly different.

At baseline, patients were treated with either human insulin mix 50 or human insulin mix 30/70, at means of 0.316 units/kg for the morning dose and 0.272 units/kg for the evening dose. At study end-point, patients were administered means of 0.364 units/kg morning dose and a 0.336 units/kg evening dose for both treatment groups. The mean total dose administered at each visit slightly increased throughout the study in both treatment groups (0.663 units/kg to 0.699 units/kg for LM50 and 0.667 units/kg to 0.708 units/kg for human insulin mix 50).

### Safety

Insulin lispro mix 50 was generally well tolerated by patients treated for 3 months. No death was reported during this study. Three patients experienced one serious adverse event (SAE) each causing their hospitalisation. One patient during LM50 treatment experienced pneumonia, while two patients during human insulin mix 50 treatment experienced coronary artery disease and hepatitis E respectively. All three SAEs were regarded by the study investigators to have no relationship with either the study drug or device. Three patients in human insulin mix 50 treatment discontinued from this study because of adverse events of hepatitis E, hepatic cirrhosis and angioneurotic oedema respectively. No patient in LM50 treatment discontinued from this study because of adverse events.

Similar numbers of patients experienced at least one treatment-emergent adverse event (TEAE) in each treatment group (39 in LM50 and 37 in human insulin mix 50). The most common TEAEs reported by patients were nasopharyngitis followed by hyperuricaemia and then hypertension in both treatment groups. Mean laboratory results for both treatment groups at baseline and end-point were all within the normal range. The mean changes in vital signs were small and not considered clinically significant.

Twenty-seven patients experienced at least one episode of hypoglycaemia in each treatment period. No statistically significant difference was found between treatment groups for the incidence of hypoglycaemia within patient (p = 0.828) nor in the rate of hypoglycaemia per 30 days (p = 0.401).

## Discussion

This study compared the glucodynamics and safety of premix formulations of insulin lispro and ILPS in a 1 : 1 ratio (LM50) vs. human insulin mix 50, in a twice a day regimen in a Chinese population. It was important to compare these two insulin mixtures because human insulin mix 50 has been widely prescribed for patients in China, while LM50 is a newer analogue formulation. The high-carbohydrate standard test meal in this study represents a high-carbohydrate Chinese diet. With this meal, LM50 provided better control of postprandial hyperglycaemia in patients with diabetes; and during the 3-month treatments, LM50 did not increase the incidence of hypoglycaemia compared with human insulin mix 50. However, despite the improved PPBG with LM50, HbA1c means were similar between the two insulin treatments.

As with our results, Roach et al. (13) reported improved postprandial glycaemic control and similar overall glycaemic control with insulin lispro mixtures as compared with human insulin mixtures. In their study, patients received LM50 and LM25 for the morning and evening meals, respectively, as one treatment, and human insulin mix 50 and 30 for the morning and evening meals, respectively, as the other treatment, for 3 months. Contrary to the equivalent rates of hypoglycaemia between treatments in our study, they reported less nocturnal hypoglycaemia with insulin lispro mixtures. Nocturnal hypoglycaemia was not assessed in our study.

In the past, rapid-acting insulin lispro and the basal insulin ILPS have been formulated in different ratios in search of an optimal bolus-basal combination. Heise et al. (17) compared the pharmacokinetic and pharmacodynamic profiles of various mix formulations of these two insulins (i.e. 100%, 75%, 50% and 25% insulin lispro). That study suggested increased insulin serum levels were achieved with increasing proportions of insulin lispro in a linear dose-dependent manner. Similarly, Schwartz et al. (18) compared human insulin mix 30 with insulin lispro mix 25 and LM50 in a test meal after a single injection. They concluded insulin lispro mixtures were associated with better PPBG control than human insulin, and greater proportions of rapid-acting insulin lispro within the mixture were associated with better PPBG control. In another clinical trial, LM50 provided better postprandial glycaemic control after a test breakfast rich in carbohydrates compared with a similar dose of insulin lispro mix 25. Specifically, LM50 given before the standard breakfast was found to be more effective in controlling the 2-h PPBG and the 2-h PPBG excursion compared with insulin lispro mix 25 given before the same meal. The two treatments were found to be equivalent in terms of FBG, HbA1c and incidence of hypoglycaemia (19).

Our results show that 1- and 2-h PPBG excursions were significantly better controlled with LM50 when compared with those with human insulin mix 50. In addition, LM50 provided significantly lower mean 2-h, but not 1-h, PPBG. Although, there was no significant change in FBG from baseline to end-point in either treatment group, the slight increase in FBG with LM50 and slight decrease with human insulin mix 50 resulted in a statistically significant difference between groups at end-point. An explanation for this difference in FBG results could be that the effect of human insulin lasts longer than lispro and has some overlap with human insulin isophane suspension for the morning glucose.

Haemoglobin A1c was controlled equivalently with both treatments in this study. In an effort to correlate our FBG and PPBG results with HbA1c, we find guidance from a trial by Monnier et al. (3), which suggests that when HbA1c is in the range of 7.3–8.4%, FBG and PPBG contribute equally to HbA1c. In this study, the mean HbA1c values at end-point for LM50 and human insulin mix 50 were 7.59% and 7.61% respectively. So lower PPBG and higher FBG with LM50 may result in an equivalent HbA1c compared with human insulin. Thus, similar control of HbA1c with both treatments perhaps reflects the offsetting directions of PPBG and FBG between the two treatments.

No significant differences were observed between treatment groups in insulin-dose requirements of patients during the 12-week treatments. These observations are consistent with those of another 12-week trial that compared glycaemic control with fixed mixtures containing insulin lispro mix 25 vs. fixed mixtures containing human insulin mix 30 (20).

The 1 : 1 ratio of mealtime and basal insulins may not address needs associated with meals of all size and composition. To achieve adequate glycaemic control, insulins should be administered before each meal, with three times a day, which is the most common frequency. The twice-daily regimen of 1 : 1 mixtures employed here may not be appropriate for patients who consume three large meals per day.

Conclusively, LM50, well tolerated by patients over the 3-month treatment period, may be an appropriate substitute in patients who administer a human insulin mixture. This 1 : 1 ratio of insulin lispro and ILPS may provide similar HbA1c control and better PPBG control compared with human insulin mix 50 in insulin-requiring patients with type 1 or type 2 diabetes while providing the convenience of injecting immediately before meals. LM50 in a twice-a-day regimen may appeal to patients who do not want to compromise their high carbohydrate breakfast and dinner, and are willing to cut down the number of injections required per day.
